# Particles of different sizes and shapes induce neutrophil necroptosis followed by the release of neutrophil extracellular trap-like chromatin

**DOI:** 10.1038/s41598-017-15106-0

**Published:** 2017-11-03

**Authors:** Jyaysi Desai, Orestes Foresto-Neto, Mohsen Honarpisheh, Stefanie Steiger, Daigo Nakazawa, Bastian Popper, Eva Miriam Buhl, Peter Boor, Shrikant R. Mulay, Hans-Joachim Anders

**Affiliations:** 1 Medizinische Klinik und Poliklinik IV, Klinikum der Universität München, Munich, Germany; 2 Department of Anatomy and Cell Biology, Biomedical Center, Ludwig-Maximilians Universität, Munich, Germany; 3 Institut für Pathologie, Universitätsklinikum Aachen, Aachen, Germany; 4 Institute of Pathology & Department of Nephrology, University Clinic of the RWTH Aachen, Aachen, Germany

## Abstract

The human body is exposed to a wide range of particles of industrial, environmental or internal origin such as asbestos, alum, silica or crystals of urate, calcium phosphate, calcium oxalate, cystine or cholesterol. Phagocytic clearance of such particles involves neutrophils and macrophages. Here we report that neutrophils encountering such particles of diverse sizes and shapes undergo necrotic cell death, a process associated with the formation of neutrophil extracellular trap (NET)-like extracellular DNA. In human neutrophils receptor-interacting protein kinase (RIPK)-1 inhibition with necrostatin-1s or mixed lineage kinase domain-like (MLKL) inhibition with necrosulfonamide abrogated cell death and associated-neutrophil extracellular DNA release induced by all of the aforementioned particles. Similar results were obtained with *Mlkl-*deficient mice neutrophils for all particles *in vitro*. Furthermore, *Mlkl-*deficient mice lacked tophus formation upon injection of MSU crystals into subcutaneous air pouches. These findings imply that nano- or microparticle-induced neutrophil extracellular DNA release is the consequence of neutrophil necroptosis, a regulated form of cell necrosis defined by RIPK1-RIPK3-MLKL signaling. Interestingly, this finding was consistent across different particle sizes and shapes. The RIPK1-RIPK3-MLKL signaling pathway may represent a potential therapeutic target in nano- or microparticle-related diseases (crystallopathies).

## Introduction

The human body is frequently exposed to microparticles from different sources. Air-borne particulates, smoking, and occupational dusts enter the respiratory tract and expose bronchial and lung cells. Cosmetic products and certain drug carriers are enriched with industrial particles that reach external and internal epithelial barriers. In addition, microparticles, fibrils or crystals form from misfolded proteins inside the body. Finally, minerals or intrinsic metabolites form crystals upon supersaturation in excretory organs such as the biliary, salivary or urinary tracts. Usually, phagocytes clear extracellular material by phagocytic uptake and lysosomal digestion, a process that may fail, if such particles are indigestible or whenever size or shape do not allow phagocytic uptake. The consequence of clearance failure of particles is tissue inflammation and of needle-shaped or large particles is foreign body granuloma formation presenting as acute or chronic ‘crystallopathies’, respectively^1^.

Neutrophils are the most abundant phagocytes in the blood and patrol through all tissues to clear them from bacteria, cell debris or other microparticles. Bacterial killing can involve direct phagocytosis or neutrophil extracellular trap (NET) formation, a process that immobilizes bacteria via expulsion of nuclear and mitochondrial DNA, kills bacteria by the release of histones and cytoplasmic proteases, and awaits phagocytic removal of NETs by macrophages^2^. NET formation also occurs upon non-infectious stimuli and is massively involved in the pathogenesis of thrombosis, ANCA vasculitis, and crystallopathies such as gout. We recently reported that NET formation occurs when gout-related monosodium urate (MSU) crystals trigger human and mouse neutrophils to undergo RIPK1-RIPK3-MLKL-mediated necroptosis^3^. Interestingly, NET formation was also reported to occur during osmotic lysis of neutrophils^4^ challenging the concept of “suicidal NETosis”. Data from another group questioned neutrophil necroptosis, albeit using different experimental conditions, not validating *in vitro* findings *in vivo*, and not at all addressing the aspect of crystal-induced DNA release^5^. To further explore the possibility whether crystalline particles induce NET-like extracellular DNA release along neutrophil necroptosis, we exposed neutrophils to a wider range of crystallopathies-related pathogenic particles. We hypothesized that particles of various sizes and shapes would all trigger RIPK1-RIPK3-MLKL-dependent neutrophil necroptosis and whether this form of regulated necrosis in neutrophils is associated with NET-like extracellular DNA release *in vitro* and *in vivo*.

## Results

### Particles of various sizes and shapes induce NET-like extracellular DNA release

**Figure 1:**
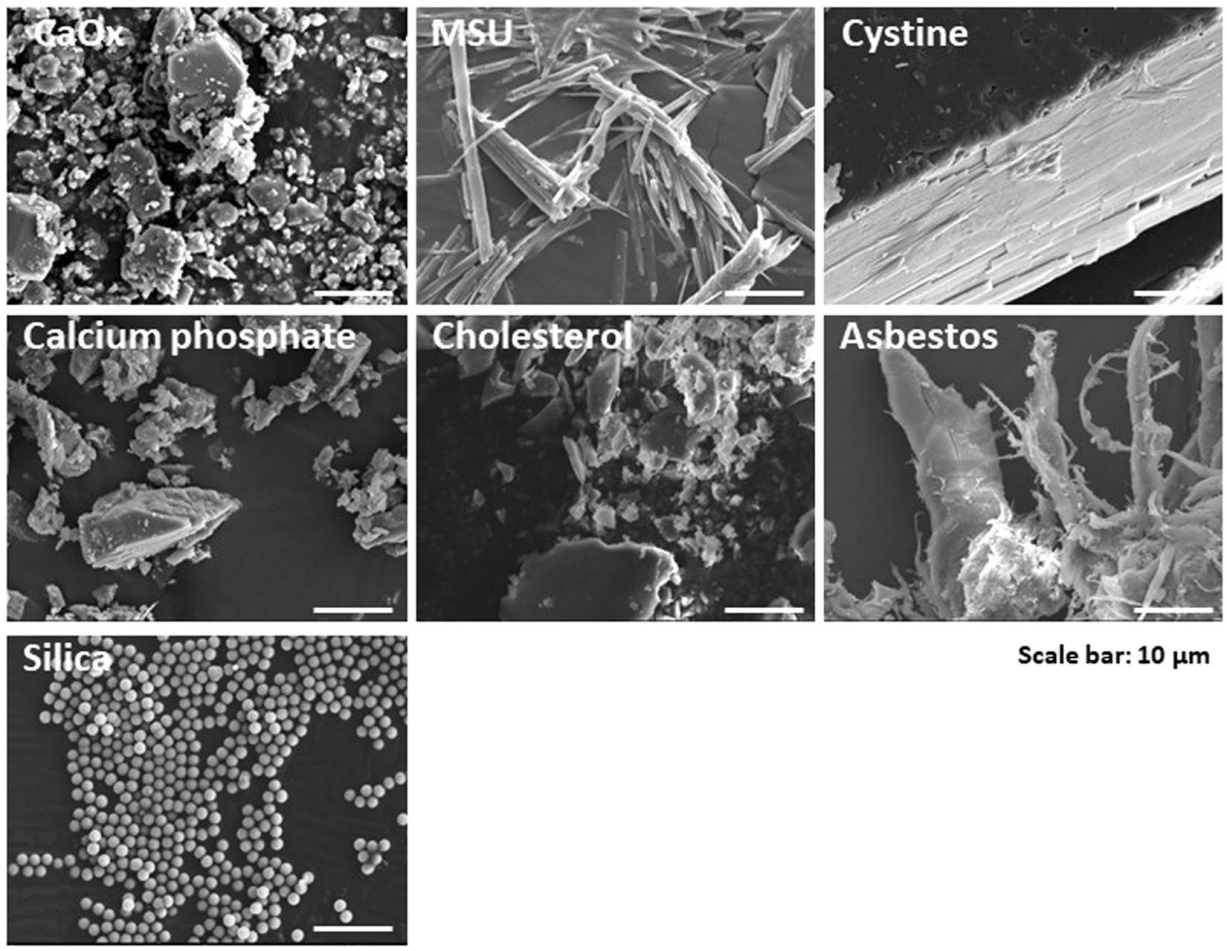
Crystals have different sizes and shapes. Crystals of calcium oxalate (CaOx), monosodium urate (MSU), cystine, calcium phosphate, cholesterol, asbestos, and silica were imaged using scanning electron microscope (scale bar = 10 µm).

**Figure 2:**
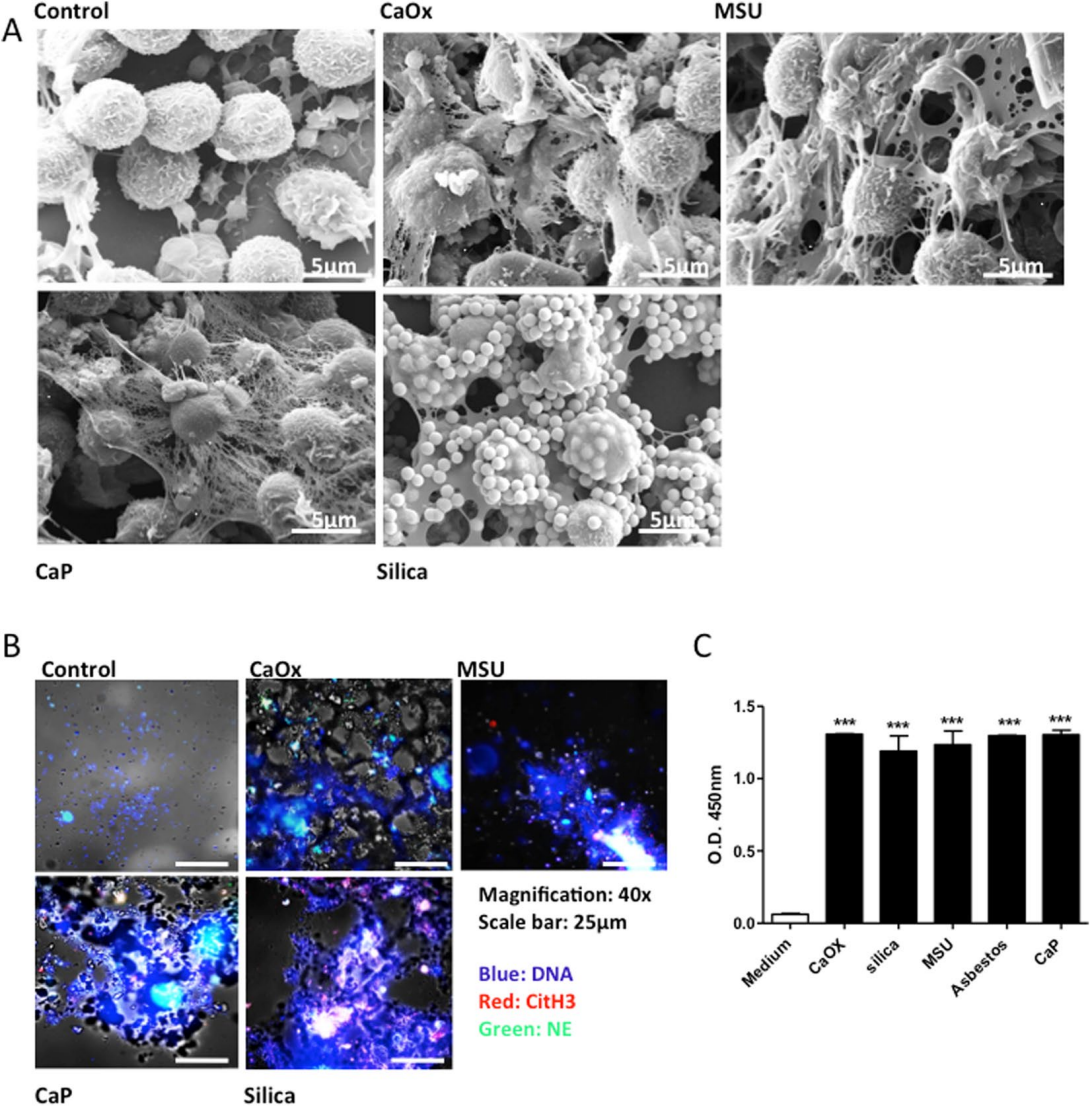
Different crystals induce NET formation. Human neutrophils were exposed to crystals of calcium oxalate (CaOx) (0.2 mg/ml), MSU (0.2 mg/ml), calcium phosphate (0.2 mg/ml), and silica (0.2 mg/ml) for 2 hours. Scanning electron microscopy was used to visualize crystal associated NETs (scale bar = 5 µm). Please note some platelets alongside the neutrophils in the control image. Arrows indicate the presence of NETs and NET-crystal aggregates. (**A**) Neutrophils exposed to crystals were co-stained for NET markers DNA (blue), citrullinated histones H3 (red) and neutrophil elastase (green). Cells were imaged using a fluorescence microscope. Crystals can be seen in phase contrast (grey) (magnification 40x). (**B**) NETs were quantified by MPO-DNA complex ELISA after 2 hours of crystal exposure (**C**). All representative images are from a single experiment. Data were obtained from three independent experiments each performed in duplicate. Data represent means ± SEM. ***p < 0.001 versus medium.

To test our hypothesis we applied a series of different particles including crystals of calcium oxalate monohydrate (CaOx), MSU, cysteine, calcium phosphate (CaP), cholesterol, and silicon dioxide (SiO_2_, silica). Furthermore, we also examined crocidolite asbestos fibers. Figure 1 illustrates the different sizes and shapes of these particles as captured by scanning electron microscopy. We isolated human neutrophils from healthy blood donors at a purity of >95% (Supplementary Figure 1). Exposing such neutrophils to each of these particles for 2 hours induced aggregated-NET-like extracellular DNA relase formed by the particles, intact neutrophils, dead neutrophil debris, and their respective extracellular chromatin (Fig. 2A, Supplementary Figure 2A,B). NET formation became obvious by phase contrast-fluorescent microscopy illustrating extracellular nuclear DNA co-localizing with citrullinated histone H3 and cytoplasmic proteases such as elastase (NE) (Fig. 2B, Supplementary Figures 2C and 3). In order to validate NET formation with another specific method, we quantified complexes of myeloperoxidase (originating from the neutrophil cytoplasmic granules) with DNA (originating either from nuclei or mitochondria) by sandwich ELISA in culture supernatants collected after 2 hours of crystal exposure to neutrophils. All particles significantly increased the concentration of such complexes in the cell culture supernatant as compared to medium control (Fig. 2C). We conclude that particles of various sizes and shapes induce neutrophils to form crystal/NET aggregates containing neutrophils extracellular DNA.

### Particles of various sizes and shapes induce neutrophil necrosis

**Figure 3:**
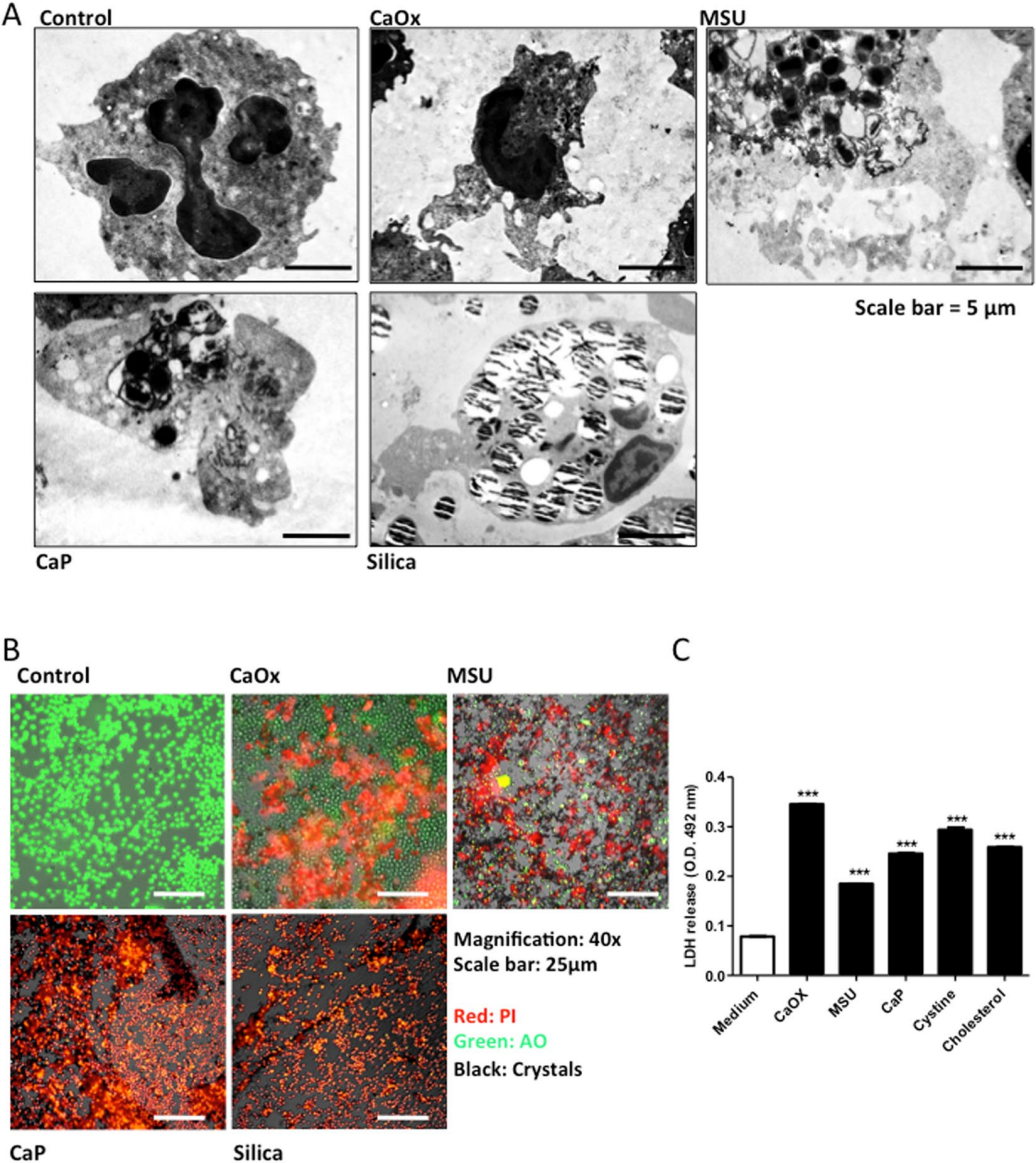
Different crystals induce neutrophil cell death. Human neutrophils were exposed to crystals of calcium oxalate (0.2 mg/ml), MSU (0.2 mg/ml), calcium phosphate (0.2 mg/ml), and silica (0.2 mg/ml) for 2 hours. Transmission electron microscopy was used to visualize crystal-associated neutrophil cell-death (scale bar = 5 µm) (**A**). Crystal-induced cell death was visualized by live cell imaging using propidium iodide (PI, red area). Acridine orange (AO) stained live cells. Crystals were imaged in phase contrast (grey) (magnification 20x) (**B**). LDH release was measured in the supernatants of crystal-exposed neutrophils post 2 hours (**C**). All representative images are from a single experiment. Data were obtained from three independent experiments each performed in duplicate. Data represent means ± SEM. **p < 0.01, ***p < 0.001 versus medium.

NET formation can or cannot be associated with neutrophil necrosis. To test whether the aforementioned particles induce NET-like releases in association with neutrophil necrosis we studied the ultrastructure of neutrophils exposed to different crystals after 2 hours. Transmission electron microscopy revealed loss of nuclear segmentation, chromatin decondensation, plasma membrane rupture and release of intracellular material in the extracellular space of neutrophils exposed to particles but not in controls (Fig. 3A). Furthermore, we employed a live cell imaging approach using acridine orange and propidium iodide (AO-PI) to distinguish live vs. necrotic neutrophils. Exposing neutrophils with crystals induced neutrophil necrosis (Fig. 3B, Supplementary Figure 4). Since, PI can bind to both DNA in cells with permeable membrane or to extracellular DNA and AO can bind to DNA, as well as RNA, we further quantified the lactase dehydrogenase (LDH) concentrations as a marker of plasma membrane rupture in supernatants of crystals-exposed neutrophils. All particles induced a significant increase of LDH release from neutrophils (Fig. 3C). Thus, particles of various sizes and shapes induce neutrophil extracellular DNA release in association with of neutrophil necrosis.

### Targeting RIPK1 and MLKL suppresses particle-induced necrosis and NET-like formation in human neutrophils

**Figure 4:**
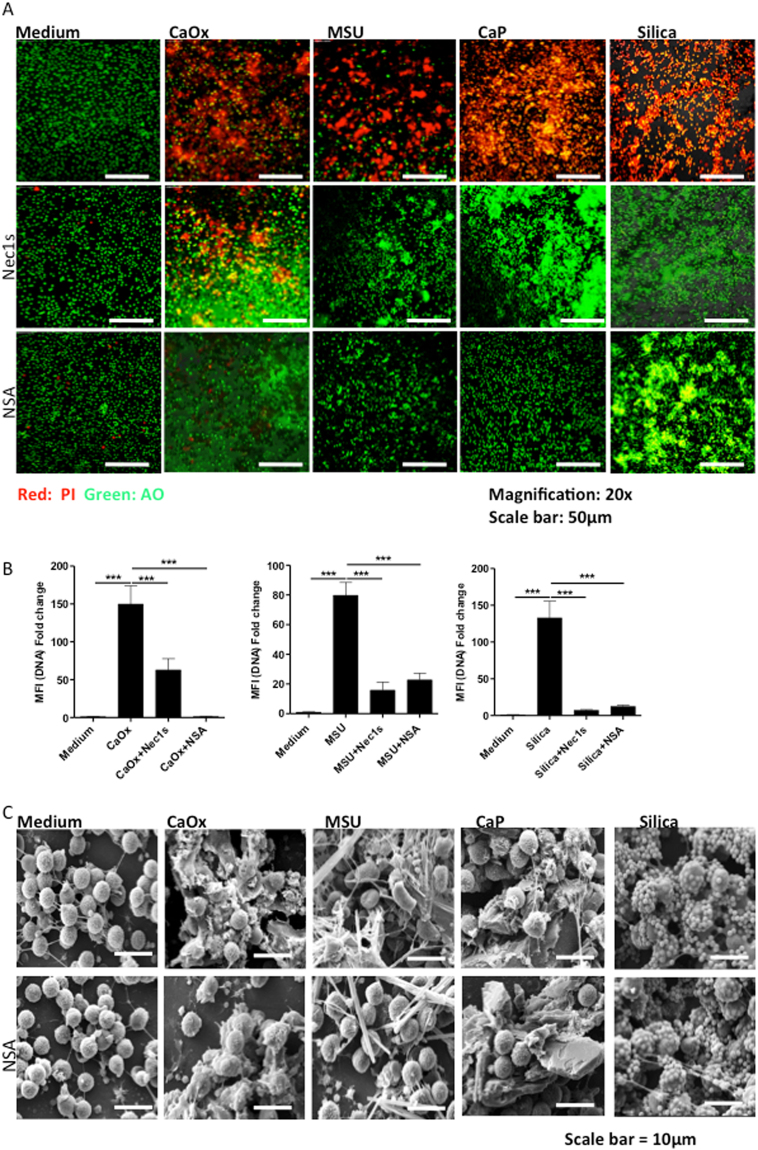
Different crystal induced neutrophil cell death involves neutrophil necroptosis. Human neutrophils were exposed to crystals of calcium oxalate (0.2 mg/ml), MSU (0.2 mg/ml), calcium phosphate (0.2 mg/ml), and silica (0.2 mg/ml) for 2 hours after 30 minutes pretreatment with 100 µM Nec1s or 5 µM NSA. Crystal-induced cell death was visualized by live cell imaging using propidium iodide stain (PI, red area). Acridine orange (AO) stained live cells (magnification 20x) (**A**) and mean fluorescence intensity was quantified (**B**). All representative images are from a single experiment. Data were obtained from three independent experiments each performed in duplicate. Data represent means ± SEM. *p < 0.05, **p < 0.01, ***p < 0.001 versus medium. After 30 minutes pretreatment with NSA (5 µM), neutrophils exposed to crytsals were fixed and visualized using scanning electron microscope (scale bar = 10 µm) (**C**).

Cell necrosis can occur as a passive or regulated process^6^. Based on our earlier observations we speculated that nano- and micro-particles might induce neutrophil necrosis involving RIPK1/RIPL3/MLKL-dependent necroptosis^3^. We used specific inhibitors of this necroptosis pathway, i.e. necrostatin 1s (blocking RIPK1 activity in necroptosis) and necrosulfonamide (blocking MLKL-mediated necrosome formation) and applied live cell imaging with the cell viability marker (AO-PI) to distinguish live from death neutrophils. Both inhibitors of necroptosis partially suppressed neutrophil death induced by all aforementioned particles (Fig. 4A,B, Supplementary Figure 5A,B). Furthermore, scanning electron microscopy studies confirmed that necrosulfonamide inhibits the formation of NET-crystal aggregates (Fig. 4c, Supplementary Figure 5). We conclude that the necroptosis-signaling pathway is involved in necrosis and the associated NET-like extracellular DNA release of human neutrophils induced by particles of different sizes or shapes.

### Mouse neutrophils deficient in MLKL lack NET-like extracellular DNA release upon exposure to particles

**Figure 5:**
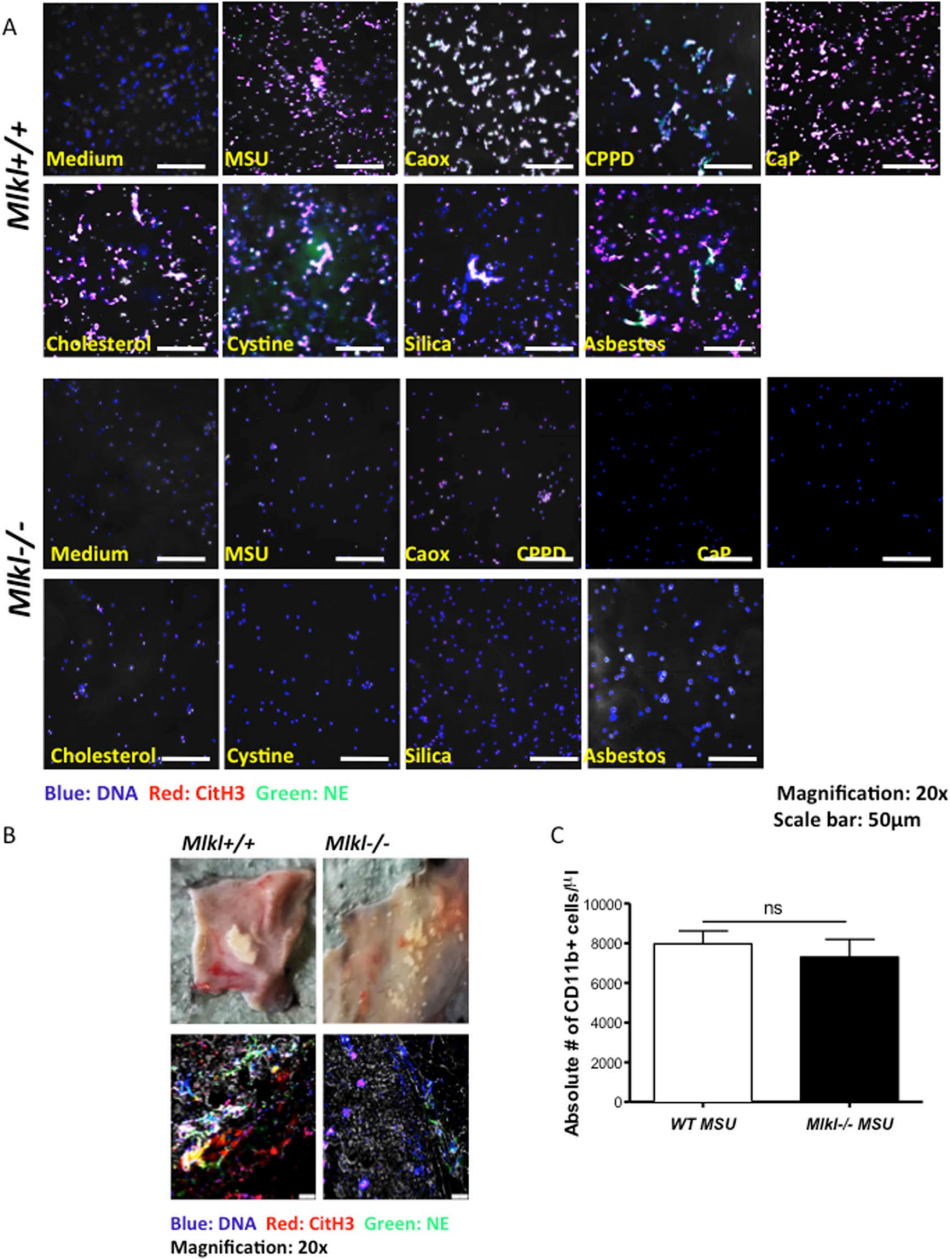
MLKL deficiency abrogates crystal-induced neutrophil cell death. Murine neutrophils from C57/BL6 (WT) and *Mlkl*−/− mice were exposed to calcium oxalate (CaOx) (0.2 mg/ml), MSU (0.2 mg/ml), asbestos (0.2 mg/ml), and silica (0.2 mg/ml) for 2 hours and were co-stained for NET markers DNA (blue), citrullinated histones H3 (red) and neutrophil elastase (green). Cells were imaged using a confocal fluorescence microscope (magnification 20x) (**A**). Air pouch membranes of mice 24 hours after injection of 2.5 mg of MSU crystals. Note the tophus-like mass (arrow), which is not present in *Mlkl*−/− mice. Air pouch membranes were stained with DNA (blue), citrullinated histones H3 (red) and neutrophil elastase (green). Representative images are shown at an original magnification of 20x (**B**). Neutrophils in the air pouch fluid were quantified 24 hours after MSU crystal injection in *Mlkl* + / + and *Mlkl*−/− mice by flow cytometry (**C**). Data represent means ± SD. ns = not significant.

We cannot fully exclude that drugs like necrostatin-1s or necrosulfonamide may modulate neutrophil viability also by yet unknown unspecific off-target effects. Therefore, to validate the role of necroptosis in crystal-induced neutrophil necrosis and NET-like extracellular DNA release we isolated neutrophils from mice deficient in MLKL or respective wild type control mice. Furthermore, NET formation was determined by fluorescent co-staining of the NET markers extracellular DNA, citrullinated histone H3, and neutrophil elastase. Crystals were imaged using phase contrast. Lack of MLKL drastically reduced extracellular DNA release and NET formation upon stimulation with all particles further confirming the involvement of necroptosis in crystal-induced neutrophil NET-like extracellular DNA release and necrosis (Fig. 5A).

### Mice deficient in MLKL lack tophus formation upon air pouch particle exposure

To further demonstrate the relevance of necroptosis in crystal-induced NET formation, we injected MSU crystals into air-pouches generated at the backside of wild type and *Mlkl*-deficient mice. When assessing tophus formation 24 h later we observed tophus formation in wild type mice but not in *Mlkl*−/− mice (Fig. 5B and Supplementary Figure 6). This was further confirmed by co-staining the air pouch skin for the NET markers extracellular DNA, citrullinated histone H3, and neutrophil elastase (Fig. 5C). The mouse genotype did not affect neutrophil recruitment into the air pouch as confirmed by pouch fluid flow cytometry (Fig. 5C). We conclude that the necroptosis-signaling pathway contributes to NET formation upon exposure to crystalline micro- and nanoparticles across a wide range of particle sizes and shapes.

## Discussion

We previously reported that crystalline particles induce RIPK1-RIPK3-MLKL-dependent necroptosis in epithelial cells and fibroblasts^7^. Here, we had hypothesized that particles of various sizes and shapes would trigger RIPK1-RIPK3-MLKL-dependent neutrophil necroptosis, and that this form of regulated necrosis would be associated with NET formation *in vitro* and *in vivo*. Our data support this hypothesis and document neutrophil necroptosis and NET-like extracellular DNA release upon exposure to crystals of MSU, CaOx, CaP, cholesterol, cystine as well as to silica, and asbestos in human and murine neutrophils.

Cell necrosis can occur in a passive manner due to mechanical trauma, temperature shock or charge-dependent disruption of the plasma membrane, e.g. upon exposure to extracellular histones^8^. However, tissue or cell necrosis can also involve one or several forms of regulated necrosis being defined by distinct signal pathways such as RIPK1/RIPK3/MLKL-dependent necroptosis, GPX4 depletion-dependent ferroptosis, CYPD-dependent mitochondrial membrane potential-related necrosis or caspase 1/11-dependent pyroptosis^6^. Therefore, the type of programmed cell death is no longer defined by morphological criteria but by the non-redundant dependence of a unique molecular signaling pathway. In contrast, “NETosis” may or may not be associated with neutrophil death and whether NET formation-related neutrophil death involves a unique signaling pathway remains under debate. The process of NET formation should be formally dissected from neutrophil death as chromatin decorated with cytoplasmic proteins may leak from neutrophils undergoing passive or regulated necrosis or may be expelled in a process not involving immediate neutrophil death^9^. It is a matter of fact that most techniques currently used to visualize, quantify, and document NET formation *in vitro* and *in vivo* are unable to either distinguish NET formation from neutrophil death or to distinguish different routines of neutrophil death^10,11^. Our *in vitro* studies consistently document that a wide range of particles trigger neutrophil necrosis in a RIPK1-MLKL-dependent manner, a signaling pathway defining necroptosis as the category of neutrophil necrosis. Neutrophil necroptosis has also been reported upon GM-CSF priming and adhesion molecule ligation^12^, as well as upon phagocytosis of *S. aureus*^13^. Our data confirm and extend our previous results on MSU crystals.

Indeed, NET formation is a critical component in acute and chronic gout^14^ and was recently shown to be triggered also by carbon or polysterene particles^15^. Our *in vitro* and *in vivo* data document that neutrophils and a wide range of nano- or microparticles form NET-like aggregates that build a creamy mass similar to what is known to be a tophus, e.g. in gout. This material is conceptually similar to pus where NET aggregates and bacteria form a creamy mass. While single NETs release numerous proinflammatory proteases, damage-associated molecular patterns, alarmins, and cytotoxic histones, NET aggregates can have also anti-inflammatory effects on their local microenvironment. In this process, proteases released from NET aggregates digest numerous proinflammatory mediators^16^. Thus, the potential of particles to induce neutrophil necrosis together with NET-like extracellular DNA release may elicit local necroinflammation or rather a state of more smoldering inflammation as known from acute or chronic gout, respectively^14,17^. The autoamplification of necroinflammation can contribute to the crescendo of local tissue injury up to organ failure and systemic inflammation^18^. Our data raise the possibility that these phenomena may apply also to other acute and chronic crystallopathies^19^.

In summary, particles induce NET-like extracellular DNA release downstream of neutrophil necroptosis. This implies that RIPK1, RIPK3, and MLKL could be molecular targets to limit crystal- and microparticle-related inflammation in acute crystallopathies and to potentially modulate chronic crystallopathies.

## Methods

### Human and murine neutrophil isolation

Blood was obtained from healthy donors. An informed consent was obtained from all subjects. All experimental protocols were approved by the “Ethikkommission der Medizinischen Fakultät der LMU”. All experimental protocols on blood collected from healthy subjects were carried out in accordance with the guidelines and regulations of “Ethikkommission der Medizinischen Fakultät der LMU”. Mouse blood samples were collected by retro-orbital bleeding technique, under isoflurane anesthesia, in microcentrifuge tubes containing heparin. All experimental protocols on mice were approved by the “The Regierung von Oberbayern, München, Germany”. All experimental protocols on blood collected from mice were carried out in accordance with the guidelines and regulations of “The Regierung von Oberbayern, München, Germany”. Blood samples were mixed with 1.25% of high molecular weight dextran (molecular weight 450K – 650K), and RBCs were allowed to settle at 4 °C. The upper clear yellowish leucocyte-rich layer was separated and lysed for the remaining RBCs using hypnotic lysis (ddH2O) and tonicity was maintained by adding 0.15 M KCl. Resultant leucocytes were enriched for neutrophils by gradient centrifugation with Biocoll solution (density of 0.177) (Berlin, Germany). As the neutrophils were very sensitive to the external stimuli, care was taken not to activate them by aggressive shaking and all the procedure were performed at 4 °C. No glass material was used as neutrophils tend to stick to glass surfaces. Once isolated, 0.5 × 10^6^ or 1 × 10^6^ neutrophils were suspended in 200 µl RPMI and seeded in 8 well µ slides (Ibidi, Martinsried, Germany) or 8 well chamber slides (Life science technologies, Darmstadt, Germany) and then incubated at 37 °C in 5% carbon dioxide atmosphere for 30 minutes.

### Scanning Electron Microscopy (SEM)

Human neutrophils seeded in 8 well chamber slides were exposed to (200–500 µg/ml) crystals like calcium oxalate (Alfa Aesar, Karlsruhe, Germany), MSU (Invivogen, Toulouse, France), calcium phosphate (Chem Cruz, Heidelberg, Germany), cysteine (Sigma life sciences, Germany), cholesterol (Invivogen), crocidolite asbestos (SPI-CHEM, West Chester, PA), and silica (Alfa Aesar) and were incubated at 37 °C in 5% carbon dioxide atmosphere for 2 hours. For some experiments, neutrophils were pre-treated with 5 µM necrosulfonamide (NSA, Millipore, Schwalbach, Germany) for 30 minutes. Supernatants were removed and the samples were fixed in 3% glutaraldehyde in phosphate buffered saline (PBS), washed in 0.1 M Soerensen’s phosphate buffer, dehydrated in an ascending ethanol series (30–100%) and dried in hexamethyl disilazan (Sigma-Aldrich, Steinheim, Germany). The samples were analysed using a scanning electron microscope (ESEM XL 30 FEG, FEI, Eindhoven, Netherlands) by 0,8T with acceleration voltage of 10 kV. After the EDX analysis (EDAX Genisis System) they were sputtered with a 12.5 nm gold palladium layer and analyzed in a high vacuum environment.

### Transmission Electron Microscopy (TEM)

For transmission electron microscopy, human neutrophils after being exposed to various crystals for 2 hours at 37 °C in 5% carbon dioxide atmosphere, were fixed in 2.0% paraformaldehyde/2.0% glutaraldehyde, in 0.1 M sodium phosphate buffer, pH 7.4 for 24 h, followed by 3 washes × 15 min in 0.1 M sodium phosphate buffer, pH 7.4 and distilled water. Again cells were post-fixed for TEM, in 2% O_s_O_4_ for 1 h, dehydrated in graded ethanol and embedded in epon. Ultrathin sections, stained with uranyl acetate and lead citrate, were observed in a JEOL EXII 1200 transmission electron microscope (Jeol, Tokyo, Japan) at 80 kv. KeenViewII (Olympus, Germany) digital camera used to take pictures and processed by the iTEM software package (analySISFive, Olympus, Germany).

### Fluorescence microscopy and Immunostaining

For immunofluorescence, neutrophils were fixed using 4% formaldehyde for 10 min at room temperature (RT) after removing the supernatant. The cells were washed with 1X PBS for 3 times and were stored in PBS at −20 °C. The cells prepared as described above, were incubated with the following primary antibodies) Neutrophil elastase (Santa Cruz Biotechnology, Santa Cruz, CA), citrullinated histone H3 (Cell Signaling, Danvers, MA) and DAPI for 1 h in PBS or 0.1% milk solution in room temperature. After washing, the sections were incubated with secondary antibodies guinea pig Alexa Fluor 488 (1:100, Invitrogen, Carlsbad, CA) or rabbit Cy3 (1:200, Jackson ImmunoResearch Laboratories, West Grove, PA) for 30 minutes at room temperature. For immunostaining studies, tophus-containing air pouch skin sections from each mouse were fixed in formalin (10% in PBS or Saline) overnight and processed using tissue processors and paraffin blocks were prepared. 2 μm thick paraffin-embedded sections were cut. De-paraffinization was carried out using xylene (3 * 5 min) followed by re-hydration, which was carried out by incubating the sections in 100% absolute ethanol (3 * 3 min), 95% ethanol (2 * 3 min) and 70% ethanol (1 * 3 min) followed by washing with PBS (2 * 5 min). Blocking endogenous peroxidase was carried out by incubating sections in H_2_O_2_ and methanol mixture (20 ml of 30% H202 in 180 ml of methanol) for 20 min in the dark followed by washing in PBS (2 * 5 min). For unmasking of antigen, sections were dipped in antigen unmasking solution (3 ml of antigen unmasking solution + 300 ml of distilled water) and cooked in the microwave for a total of 10 min (4 * 2.5 min, every 2.5 min water level was checked and made up to the initial levels with distilled water every time). After microwave cooking sections were cooled to room temperature for 20 min and washed with PBS. Blocking endogenous biotin was carried out by incubating sections with one drop of Avidin for 15 min, followed by incubation with Biotin (Vector Laboratories, Burlingame, California) for further 15 min. After the incubation was over sections were washed with PBS (2 * 5 min). The researcher was blinded for the groups while obtaining the images. Leica DMi8 florescence microscope was used to perform the imaging. The air objective lens 40x and 20x were used to take pictures of different fields. Multiple images were taken per group and the mean fluorescence intensity (MFI) of the stainings for different pictures was quantified using ImageJ software. Different plugins to calculate the percentage positive area compared to whole image in ImageJ were used for analysis.

### Cell death assays

Necrotic cell death was also quantified in the supernatants of crystal-exposed neutrophils using Lactate dehydrogenase (LDH) cell cytotoxicity assay kit (Roche, Mannheim, Germany) according to manufacturer’s protocol. Furthermore, live vs dead cells were analysed by fluorescence live cell imaging approach. Neutrophils after 2 hours of crystal stimulation were imaged using acridine orange (live cells) and PI stain (dead cells) (Thermo Fisher scientific, Germany). Fluorescence signals were detected using Leica fluorescence microscope (Leica, Weltzlar, Germany), and quantified using ImageJ software (National Institutes of Health, Bethesda, Maryland).

### MPO-DNA complex ELISA

Crystal-induced NETs were quantified by the MPO-DNA sandwich ELISA method using anti-DNA Abs (Roche) and anti-human MPO Abs (AbD Serotec, Raleigh, North Carolina) in supernatant using previously described method. NET-crystal aggregates were gently fragmented using pipette and were centrifuged at 1200 rpm for 2 min to settle crystals. The supernatant was then collected and used for ELISA.

### Air pouch model

The Regierung von Oberbayern, München, Germany, approved animal studies. All experiments were performed in accordance with their guidelines and regulations. Murine air pouches were generated in 6-week-old female C57BL/6 wild type and *Mlkl*−/− mice (n = 5) followed by injections of 2.5 mg MSU crystals in PBS in subcutaneous layer of the air pouch skin. 24 hours later, the air pouch fluid was harvested for neutrophil flow cytometry. Every isolate was incubated with binding buffer containing either anti-human CD11B (BD Bioscience) for 45 min at 4 °C in the dark were used to detect neutrophils. Air pouch skin with tophus was fixed with 4% paraformaldehyde and was co-stained for NET markers like DNA, citrullinated histones H3 (Cell signaling) and neutrophil elastase (Santa Cruz Biotechnology).

### Statistics

Data are presented as mean ± SEM. Unpaired Student’s t-test and One way ANOVA followed by Dunnett’s post-test were used for the comparison. A value of p < 0.05 was considered to indicate statistical significance.

## Electronic supplementary material

Supplementary information
