# Author Correction: Particles of different sizes and shapes induce neutrophil necroptosis followed by the release of neutrophil extracellular trap-like chromatin

**DOI:** 10.1038/s41598-018-24793-2

**Published:** 2018-04-24

**Authors:** Jyaysi Desai, Orestes Foresto-Neto, Mohsen Honarpisheh, Stefanie Steiger, Daigo Nakazawa, Bastian Popper, Eva Miriam Buhl, Peter Boor, Shrikant R. Mulay, Hans-Joachim Anders

**Affiliations:** 1 Medizinische Klinik und Poliklinik IV, Klinikum der Universität München, Munich, Germany; 2 Department of Anatomy and Cell Biology, Biomedical Center, Ludwig-Maximilians Universität, Munich, Germany; 3 Institut für Pathologie, Universitätsklinikum Aachen, Aachen, Germany; 4 Institute of Pathology & Department of Nephrology, University Clinic of the RWTH Aachen, Aachen, Germany

Correction to: *Scientific Reports* 10.1038/s41598-017-15106-0, published online 03 November 2017

The original version of this Article contained an incorrect version of Figure 1 containing unwanted background text. This has now been corrected in the HTML and PDF versions of the Article.

