# Supplementary material for: Particles of different sizes and shapes induce neutrophil necroptosis followed by the release of neutrophil extracellular trap-like chromatin
Source: Sci Rep. 2017 Nov 3;7:15003. doi: 10.1038/s41598-017-15106-0 (PMC5670218; doi:10.1038/s41598-017-15106-0)
Supplement: Supplementary file 1 — Supplementary information [file 41598_2017_15106_MOESM1_ESM.pdf]

Particles of different sizes and shapes induce neutrophil necroptosis followed by the release of neutrophil extracellular DNA

Jyaysi Desai[1], Orestes Foresto-Neto[1], Mohsen Honarpisheh[1], Stefanie Steiger[1], Daigo Nakazawa[1], Bastian Popper[2], Eva Miriam Buhl[3,4], Peter Boor[3], Shrikant R. Mulay[1], Hans-Joachim Anders[1]

1 Medizinische Klinik und Poliklinik IV, Klinikum der Universität München, Munich, Germany
2 Department of Anatomy and Cell Biology, Biomedical Center, Ludwig-Maximilians Universität, Munich, Germany
3 Institut für Pathologie, Universitätsklinikum Aachen, Aachen, Germany
4 Institute of Pathology & Department of Nephrology, University Clinic of the RWTH Aachen, Germany

Supplementary information

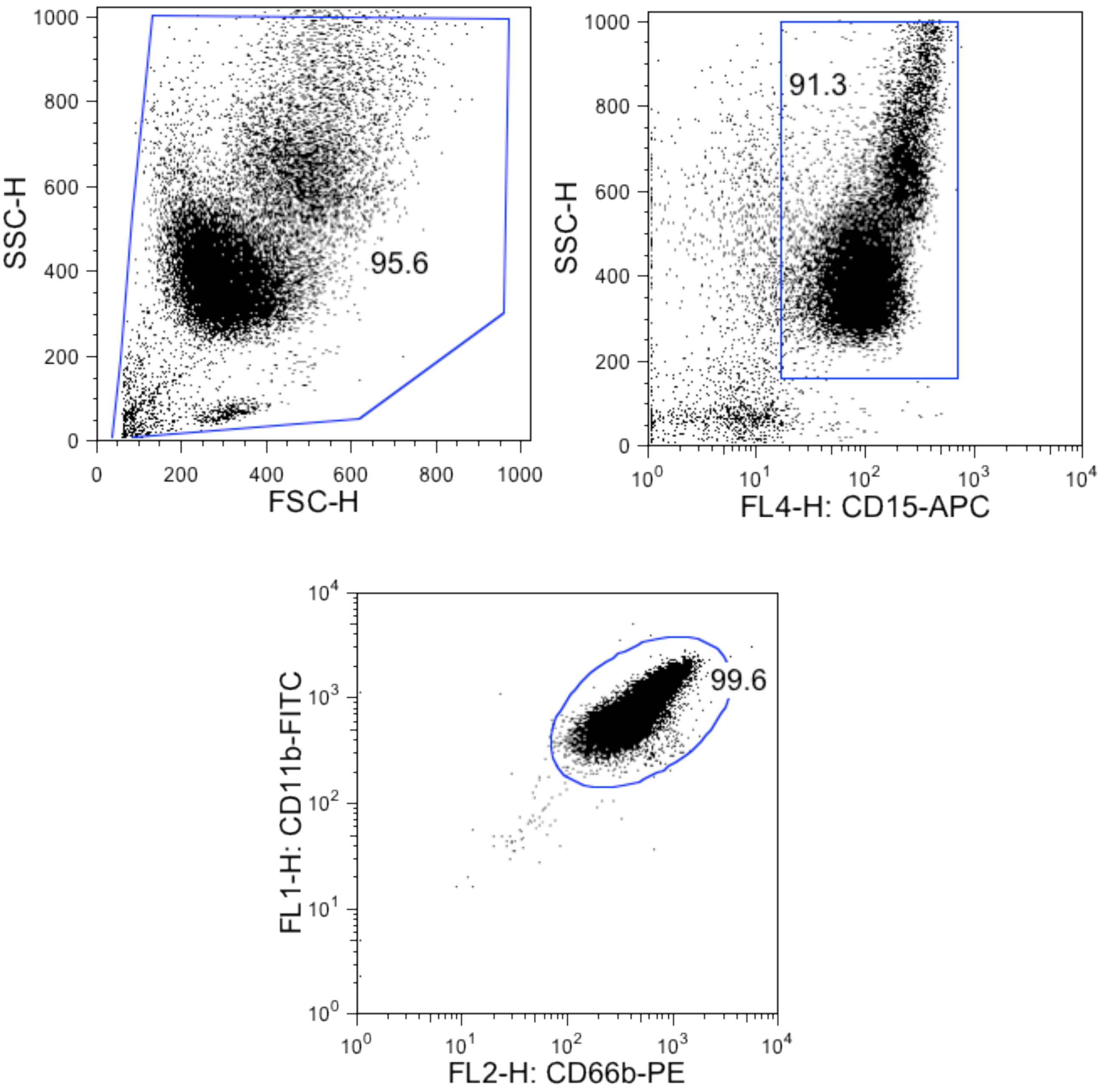

Supplementary figure 1: Flow cytometry of isolated human neutrophils**.** Human neutrophils were isolated using Ficoll density gradient. The neutrophil pellet was stained for the surface markers CD15, CD11b, and CD66b. More than 95% purity was obtained for this isolation method.

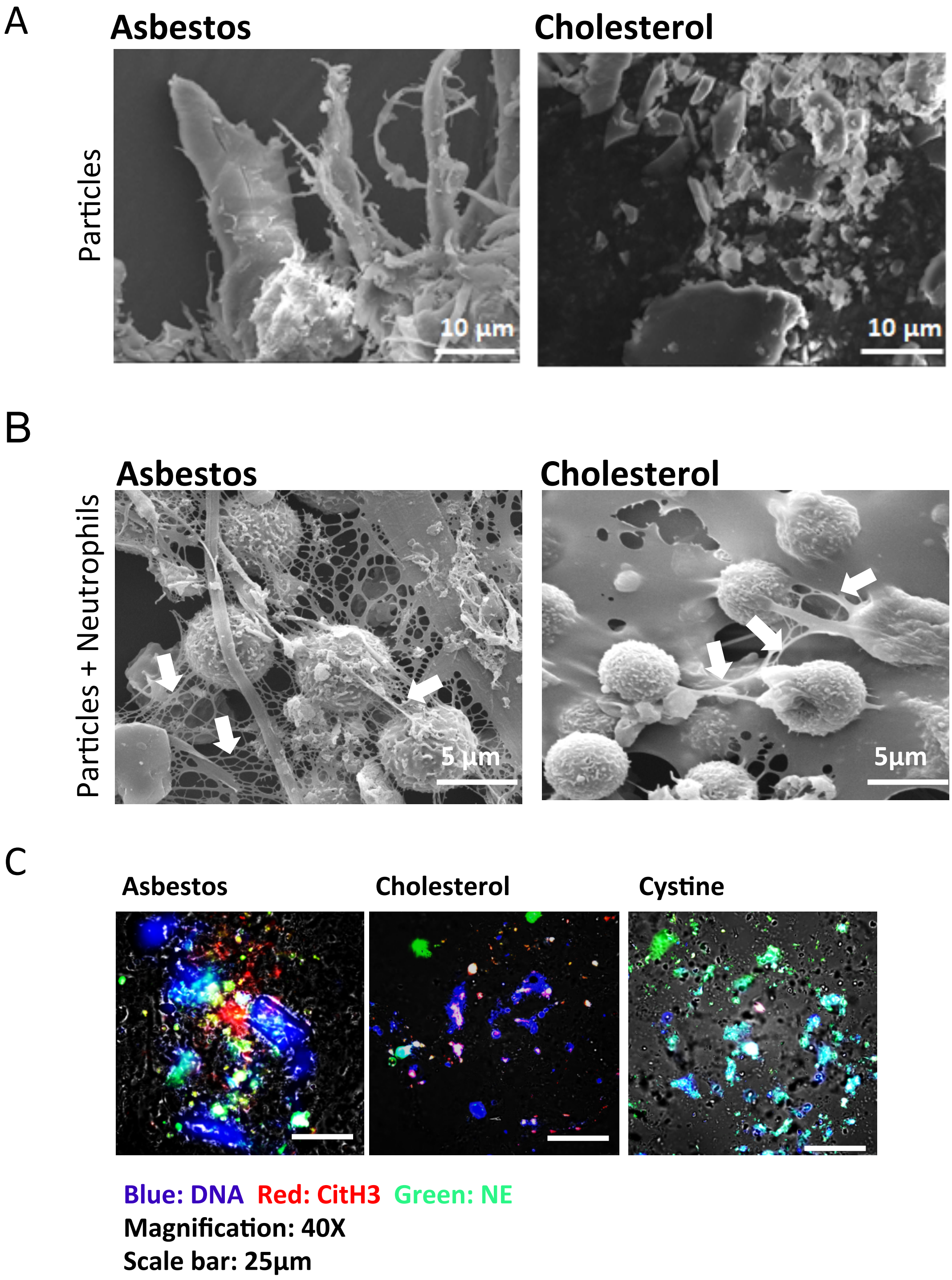

Supplementary figure 2: SEM images of particles of asbestos, and cholesterol (A). Human neutrophils were exposed to crystals of asbestos (0.2 mg/ml) and cholesterol (0.2 mg/ml) for 2 hours, were fixed and used for SEM to visualize crystal associated NETs (scale bar = 5µM). Arrows indicate the presence of NETs and NET-crystal aggregates. Please note the NET- crystal aggregates formed with TiO_2 particles (B). NETs were co-stained for NET markers DNA (blue), citrullinated histones H3 (red) and neutrophil elastase (green). Cells were imaged using fluorescence microscope. Crystals can be seen in phase contrast (grey) (magnification 40x) (B).

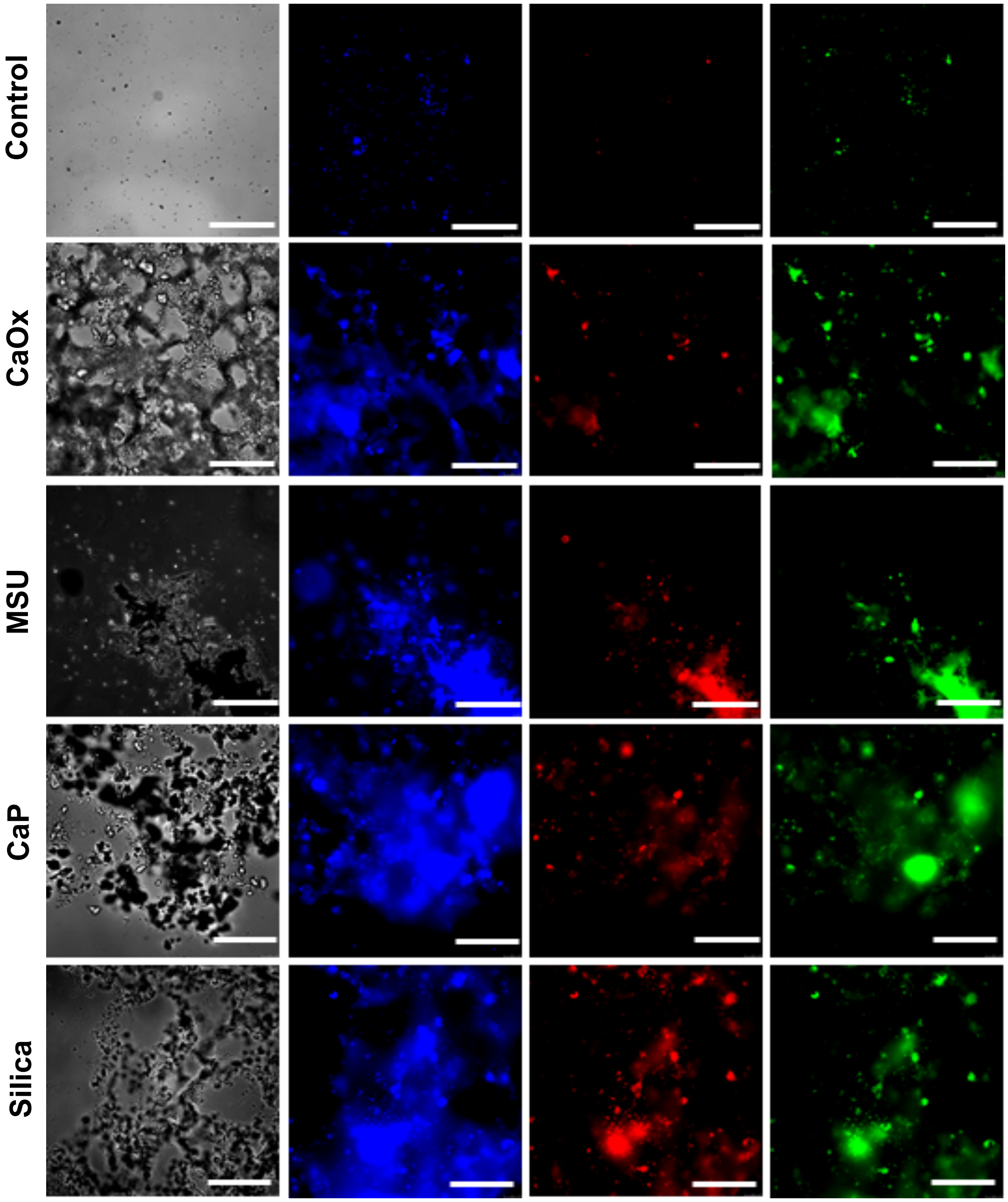

Magnification: 40X
Scale bar: 25µm

Supplementary figure 3: Human neutrophils were exposed to various crystals and NETs were co-stained for NET markers DNA (blue), citrullinated histones H3 (red) and neutrophil elastase (green). Cells were imaged using fluorescence microscope. Crystals can be seen in phase contrast (grey) (magnification 40x).

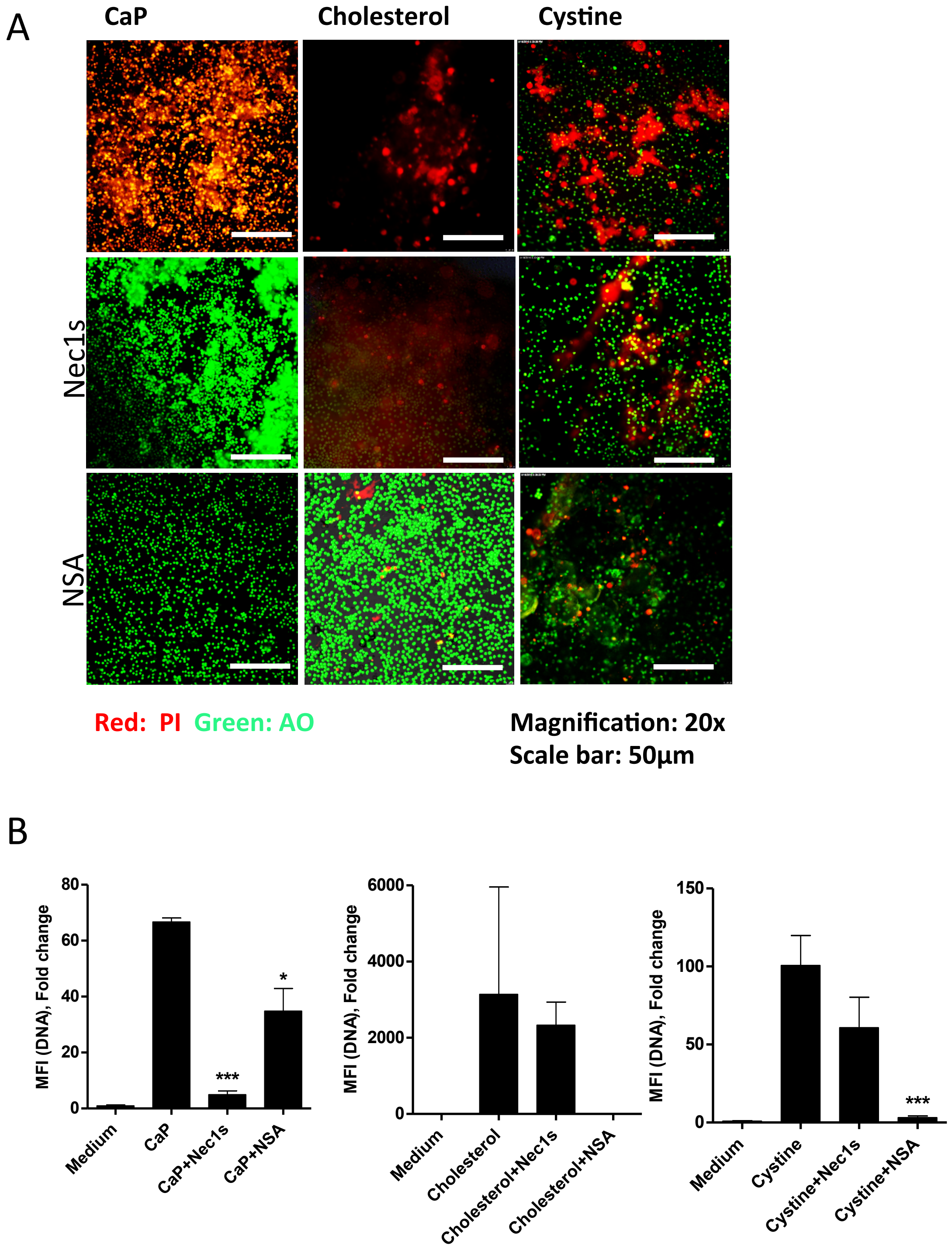

Supplementary figure 4: Human neutrophils were exposed to crystals of asbestos (0.2 mg/ml), cholesterol (0.2 mg/ml) and cystine (0.2mg/ml) for 2 hours and crystal-induced cell death was visualized by live cell imaging using propidium iodide (red area). Acryl orange (AO) stained the live cells in green (A) and were quantified (B). Data were obtained from 3 independent experiments each performed in duplicate. Data represent means ± SEM. * $p < 0.05$, *** $p < 0.001$ versus medium

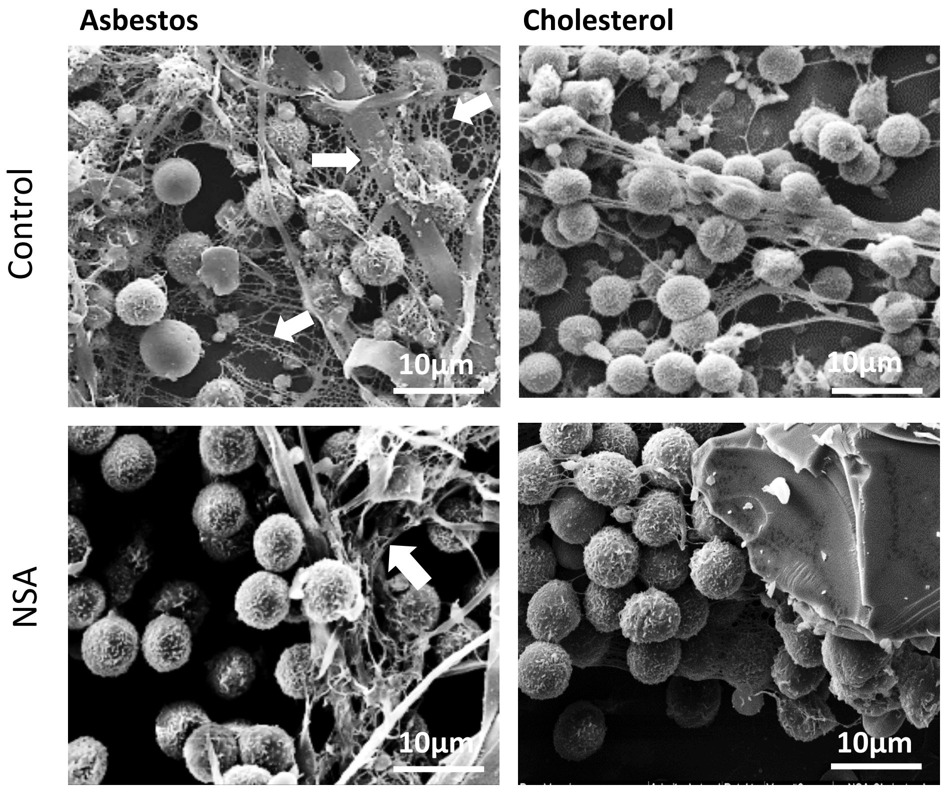

Supplementary figure 5: Human neutrophils were exposed to crystals of asbestos (0.2 mg/ml) and cholesterol (0.2 mg/ml) for 2 hours after 30 minutes pretreatment with NSA (5 µM), crystal induced neutrophils were fixed and visualized using scanning electron microscope. (scale bar = 20 µM)

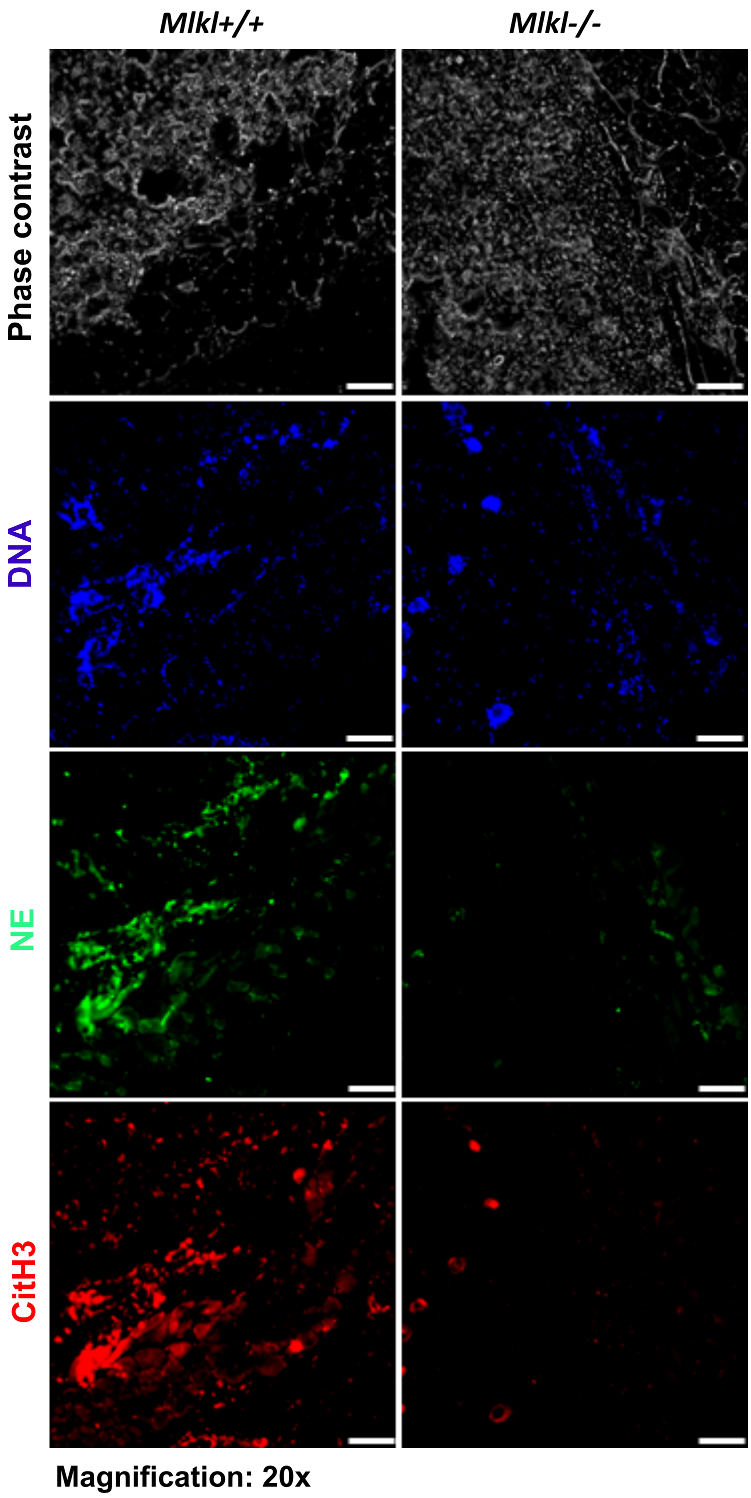

Supplementary figure 6: Air pouch membranes of mice 24 hours after injection of 2.5 mg of MSU crystals. Note the tophus-like mass (arrow), which is not present in *Mlkl*-/- mice. Air pouch membranes were stained with DNA (blue), citrullinated histones H3 (red) and neutrophil elastase (green). Representative images are shown at an original magnification of 20x.
